# Salt Reductions in Some Foods in The Netherlands: Monitoring of Food Composition and Salt Intake

**DOI:** 10.3390/nu9070791

**Published:** 2017-07-22

**Authors:** Elisabeth H. M. Temme, Marieke A. H. Hendriksen, Ivon E. J. Milder, Ido B. Toxopeus, Susanne Westenbrink, Henny A. M. Brants, Daphne L. van der A

**Affiliations:** National Institute for Public Health and the Environment (RIVM), 3720 BA Bilthoven, The Netherlands; Marieke.Hendriksen@rivm.nl (M.A.H.H.); Ivon.Milder@rivm.nl (I.E.J.M.); Ido.Toxopeus@rivm.nl (I.B.T.); Susanne.Westenbrink@rivm.nl (S.W.); Henny.Brants@rivm.nl (H.A.M.B.); Daphne.van.der.A@rivm.nl (D.L.v.d.A.)

**Keywords:** sodium, salt, food reformulation, food composition, nutritional status, 24 h urine

## Abstract

Background and objectives. High salt intake increases blood pressure and thereby the risk of chronic diseases. Food reformulation (or food product improvement) may lower the dietary intake of salt. This study describes the changes in salt contents of foods in the Dutch market over a five-year period (2011–2016) and differences in estimated salt intake over a 10-year period (2006–2015). Methods. To assess the salt contents of foods; we obtained recent data from chemical analyses and from food labels. Salt content of these foods in 2016 was compared to salt contents in the 2011 version Dutch Food Composition Database (NEVO, version 2011), and statistically tested with General Linear Models. To estimate the daily dietary salt intake in 2006, 2010, and 2015, men and women aged 19 to 70 years were recruited through random population sampling in Doetinchem, a small town located in a rural area in the eastern part of the Netherlands. The characteristics of the study population were in 2006: *n* = 317, mean age 49 years, 43% men, in 2010: *n* = 342, mean age 46 years, 45% men, and in 2015: *n* = 289, mean age 46 years, 47% men. Sodium and potassium excretion was measured in a single 24-h urine sample. All estimates were converted to a common metric: salt intake in grams per day by multiplication of sodium with a factor of 2.54. Results. In 2016 compared to 2011, the salt content in certain types of bread was on average 19 percent lower and certain types of sauce, soup, canned vegetables and legumes, and crisps had a 12 to 26 percent lower salt content. Salt content in other types of foods had not changed significantly. Between 2006, 2010 and 2015 the estimated salt intake among adults in Doetinchem remained unchanged. In 2015, the median estimated salt intake was 9.7 g per day for men and 7.4 g per day for women. As in 2006 and 2010, the estimated salt intake in 2015 exceeded the recommended maximum intake of 6 g per day set by the Dutch Health Council. Conclusion. In the Netherlands, the salt content of bread, certain sauces, soups, potato crisps, and processed legumes and vegetables have been reduced over the period 2011–2016. However, median salt intake in 2006 and 2015 remained well above the recommended intake of 6 g.

## 1. Introduction

Dietary factors such as the intake of sodium (salt) and saturated fatty acids, and the related risk factors of a high systolic blood pressure are among the leading causes of non-communicable disease [1]. The World Health Organization (WHO) Member States have agreed on a voluntary global noncommunicable diseases (NCD) target for a 30% relative reduction in mean population intake of salt, with the aim of achieving a target of less than 5 g per day (approximately 2 g sodium) by 2025 [2]. The Dutch Health Council advises a maximum intake of 6 g (2.4 g sodium) per day [3]. Intake of salt in the Netherlands and most developed countries is well above these recommended intakes [4,5,6,7]. Major sources of salt are bread, cheese, meat and meat products (including meat cold cuts), savoury snacks, sauces, soups, and pastries [5,8,9]. Reformulation of these types of foods to reduce salt contents (or food product improvement/reformulation) is considered a promising strategy for lowering the dietary intake of salt [10]. To decrease salt intake of a certain population, large-scale structural efforts are needed to lower the salt content of food products at the time of production, as well as to initiate behavioural changes [11,12,13]. The WHO encourages a multisector approach, including partnerships between the public and private sectors to improve the composition of the food supply [10].

**Figure 1:**
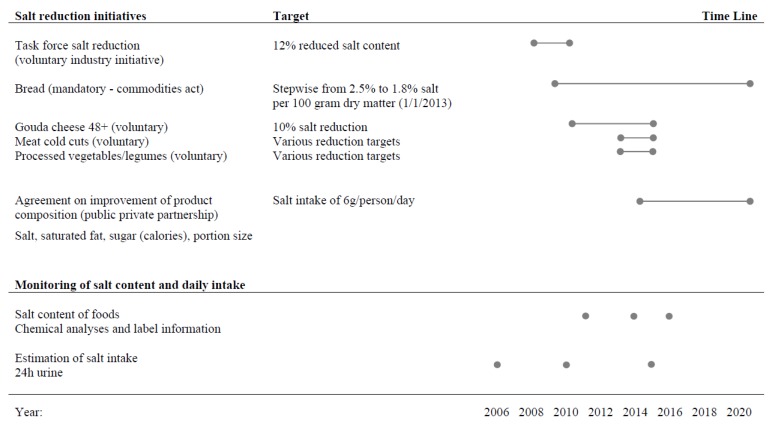
Overview of salt reduction targets and monitoring.

In the Netherlands, several initiatives focus on food product improvement related to salt (see Figure 1). The Federation of the Dutch Food and Grocery Industry initiated a Taskforce Salt Reduction in 2007. This taskforce, which included, e.g., producers of sauces and soups, cheese, snacks, and pastries, aimed at a reduction of salt levels in processed foods of 12% before 2010 [14]. The Dutch bakery sector has been an active player in the field of salt reduction via regulations laid down in the commodities act. The maximum level of salt in bread gradually decreased over the last decade [15]. In 2009, the maximum salt content per 100 g dry matter was 2.5%, in 2011 2.1%, and in 2012 1.9%. The latest amendment to the maximum level was on 1 January, 2013, at 1.8% per 100 g dry matter. Based on an average dry matter content of 64%, this is approximately 1.15 g per 100 g of bread (454 mg of sodium). Monitoring reports of the sector using analytical methods showed the expected reductions in salt contents of bread [16,17]. In 2014, the Agreement on Improvement of Product Composition: salt, saturated fat, and sugar (calories) was signed with four food sector parties under the supervision of the Dutch Ministry of Health, Welfare and Sport [18]. In this public-private agreement, several stakeholders involved in food production, hospitality, catering and retail were represented. Signing parties voluntarily agreed on a gradual reduction of the levels of salt, (saturated) fat and energy (from sugar and fat) in foods up to 2020. For salt, the ultimate goal is to reduce salt intake to 6 g/day by 2020, for diets complying with healthy dietary guidelines.

The National Institute of Public Health and the Environment (RIVM) is commissioned by the Ministry of Health, Welfare and Sport to monitor the changes in nutrient composition of foods sold in retail [8,19], as well as to monitor the effects on the intake in the population. Effects on daily dietary salt intake can be assessed by measuring salt excretion in 24 h urine. Estimation of salt intake from 24 h urinary sodium excretion is a more reliable method than dietary assessment because of the highly variable sodium content in recipes and the difficulty to quantify discretionary salt used in cooking and at the table.

This study has two objectives. First, it describes salt contents of foods in the Dutch market over a 5-year period (2011–2016) and secondly it reports on the results of monitoring salt intake in the Dutch population over a 10-year period (2006–2015 using 24 h urine collections).

### 2.1. Evaluation of Salt Content of Foods

In this study, we evaluated the current salt content of foods compared to a reference. Food composition as provided in the Dutch Food Composition Database (NEVO, version 2011; [20]) was taken as a reference. New food composition data were collected until the end of June 2016. The average salt contents (as well as concentrations of saturated fatty acids and mono-and disaccharides) were calculated. Sodium levels (in grams) were multiplied by 2.54 to calculate the salt contents of foods (in grams). Salt contents include both naturally occurring as well as added salt. Two analyses were performed to evaluate the salt contents: one on the main food categories, and one on the foods with set salt maximum levels as defined in the Agreement on Improvement of Product Composition. This was done to evaluate whether achieved reductions for specific foods under the agreement also led to lower salt concentrations in the overall food category.

#### 2.1.1. Selecting Major Processed Foods Contributing to Salt Intake

The major processed foods contributors to dietary salt intake were selected based on the salt content available in the Dutch Food Composition Database (NEVO), combined with consumed quantities of foods from the national Food Consumption Survey [5].

We included:

- Foods that can be reformulated for their salt content. For example, the food group “milk products” is excluded from the salt analyses, because the foods in this group do not contain added salt, and thus reformulation for salt is not feasible.
- Food groups that contributed at least 3% and food subgroups that contributed at least 0.5% to the daily intake of salt intake according to the Dutch National Food Consumption Survey for 7–69 year-olds (DNFCS 2007−2010); [5] and/or foods with a set maximum level for salt [21].
- Food groups with at least 10 comparable foods in the newly collected food composition data.

The food composition was compared for foods categorized in food groups. The food group categorization used consisted up to five hierarchical levels. The food group classification was based on the food group classification used in the Dutch food-based dietary guidelines (in Dutch: Wheel of Five in 2011) [22]. After consultation with the food industry, modifications were made to improve correspondence with food group classifications used by the food industry.

Foods were classified into food groups based on name and nutritional values by experienced dieticians.

The Dutch Food Composition Database (NEVO, version 2011; [20]) provided the reference values for the current comparison. The Dutch food composition database contains data, on an aggregated level, on the composition of foods and dishes eaten frequently by the Dutch population (based on the National Food Consumption Survey) and of major foods of importance for energy and/or selected nutrients. It provides food composition data for generic foods when possible. This means that data from comparable foods (for instance semi-skimmed milk of various brands) is aggregated to give a weighted mean value for the generic food (for instance ‘semi-skimmed milk’). When aggregation is not possible, for instance for fortified foods, or foods listed under their brand name, the aggregated nutrient values are derived from chemical analyses, from label information, from recipe calculations, or other sources. For the reference dataset, we used (aggregated) nutrient values only when derived from (chemical) analyses and/or information obtained from the manufacturer. For food groups for which the food composition database contained insufficient data to construct a reference value, data were supplemented with data from the Innova database [23]. The Innova database contains label information on new foods on the market. A login account is needed to access the database.

#### 2.1.2. Salt Content of Selected Processed Foods; New Data Collection and Selection

The salt contents referred to the unprepared products as sold; with the exception of some instant soups and instant sauces (sold as a dried powder). Thus, salt added during (home) preparation as well as at the table was not taken into account. For soups and sauces sold as dried powder, the salt content was calculated using the standard method of preparation.

New data collection took place until 1 July 2016. Data were mainly obtained from two sources, chemical analytical data by the Dutch Food Safety Authority (NVWA) and label information from the Food Label Database [24]. In addition, the Dutch Bakery Association provided chemical analytical data.

The NVWA carries out chemical analyses of salt in various foods. For this monitoring study commonly eaten foods were sampled (in total *n* = 1108 samples) in several major supermarkets (including private and national supermarket brands). The NVWA supplied data on for bread (*n* = 88), savoury snacks (*n* = 197), sauces (*n* = 88), soups (*n* = 102), confectionary and bakery ware (*n* = 92) and processed vegetables and legumes (*n* = 106) in 2015 [25]. In 2016 [26,27], salt contents of meats (*n* = 247), cheese (*n* = 98), sauces (including ketchup, curry and pasta sauces) (*n* = 10), and ready meals (*n* = 80) were sampled. Sodium contents were analysed by flame emission spectroscopy. For analysis, 350 mg ± 10 mg homogenized sample was weighed in a quartz vial insert. Three millilitres of nitric acid were added and the quartz vial was weighed without the cover (mass A). Then, the quartz insert keg was placed in a pressure vessel with 6 mL of 12.5% H_2_O_2_ solution. The pressure vessel was placed in a microwave with a PC-controlled temperature program (model Ethos one; Milestone Inc., Shelton, CT, USA) and digested according to the following program: 0 min, 20 °C; 0–15 min, 20–200 °C; 15–40 min, 200 °C; 40–60 min, 200–20 °C. The quartz insert keg was taken out of the pressure vessel by tweezers, cooled down by rinsing the outside with demineralised water, and dried with a tissue. Subsequently, the quartz keg was weighed and brought to mass A again with nitric acid. The digested sample was quantitatively transferred to a 50 mL plastic tube and made up to a final weight of 51.30 g (±0.05 g) with demineralised water. Two millilitres of the resulting solution was transferred to a plastic tube, diluted 10-fold with 18 mL of demineralised water, and thoroughly mixed prior to analysis. The solution was further diluted with a 0.39% nitric acid solution if necessary. Sodium was analysed by a flame photometer (Model 420 Flame Photometer; Sherwood Scientific Ltd., Cambridge, UK) with a propane/butane flame at a wavelength of 589 nm. Prior to analysis, the spectrophotometer was stabilized for 30 min with demineralised water. A calibration curve was built in the range 0.00–5.00 mg L^−1^ (six standard solutions). A 0.39% nitric acid solution was used as blank. The method was validated according to standard ISO 17025 guidelines with the following certified reference materials: LGC 7103 (sweet biscuits) and BCR 063R (skimmed milk powder). The method of performance characteristics were as follows: Recovery values: 102.1% for biscuits and 92.8% for skimmed milk powder; coefficient of variation of repeatability (RSDr): 3.0% for sweet biscuits and 0.4% for skimmed milk powder. Limit of detection (LOD): 0.23 g kg^−1^; limit of quantification (LOQ): 0.46 g kg^−1^. For the calculation of sodium chloride content, the amount of sodium was multiplied by 2.541 × 10^−4^ and the sodium chloride was expressed as g NaCl 100 g^−1^ (NaCl%). A single measurement was carried out in each sample.

The Dutch Bakery Association [16] supplied data from the seventh nationwide sample of bread, the average salt content in whole wheat bread (average of 93 breads), brown wheat bread (average of 91 breads), multi-grain bread (average of 91 breads), soft white bread buns (average of 48 buns), and soft brown bread buns (average of 19 buns). The average contents were weighted for the share industrial and artisan bread (80/20%).

New data label data was extracted from the Food Label Database [24] (*n* = 3524) (not open access) provided that the following criteria were met:

- The name and/or description of the food was clear enough to allow categorization (based on expert judgement).
- The food was aimed at individual consumers (contrary to foods for catering, clinical use, and bulk-sales).
- Foods were unique (identical foods with different packaging size were included only once).
- Data was available for on the amount of salt and/or sodium in the food.

To assess the (non)representativeness of the new data for main brands, the presence of supermarket and/or major private brands were evaluated and reported in the Table 1 To be assigned as not representative for supermarket brands (superscript a in Table 1) less than half of the major supermarkets brands (Albert Heijn, Jumbo, Aldi, Lidl, Plus, as well as at least one other supermarket brand within the Superunie purchasing organization (besides Plus) were available in the underlying data. To assess the main private brands within a food category, we listed the (up to 10) most commonly reported brands (excluding the supermarket brands) based on the national food consumption survey [5]. Presence in less than half of the intended brands was interpreted as an indication that the newly submitted data were not representative (in addition to the supermarket brands) of the product range within the relevant food category (superscript b in Table 1).

#### 2.1.3. Statistical Analyses

For each included food group (see Table 1), as well as for foods with maximum set salt levels (see Table 2), we calculated the mean salt level, and their standard deviation. The mean values were compared in an analysis of variance (*p* < 0.05). When a significant difference was found post-hoc, least squares means tests were done. All analyses were performed for men and women separately. In addition, the analyses were adjusted for age, education, and day of the week (because of overrepresentation of weekend days).

All statistical analyses were performed with the GLM procedure in SAS 9.3^®^, SAS Institute Inc, Cary, NC, USA.

### 2.2. Estimation of Salt Intake Via 24 h Urinary Sodium Excretion

#### 2.2.1. Study Population, Recruitment

In 2006, 2010 and 2015, monitoring surveys were carried out among adults aged 19−70 years in Doetinchem, a town in the eastern part of the Netherlands. The methodology of the surveys in 2006 and 2010 has been previously described in detail [4]. In 2015, a similar methodology was used.

In short, participants aged 50–70 years were recruited from an ongoing long-term monitoring study on chronic disease factors (Doetinchem Cohort Study; DCS)., and participants aged 19–49 years were recruited from the municipal register of Doetinchem (General Doetinchem Population Sample (GDPS)). Those participating in 2006 or 2010 were not invited to take part in the 2015 survey, to ensure independent study samples. Invitations were sent to a random sample of the populations, stratified for age. We aimed for equal sample size as in 2010 (*n* = 350) [4]. The current study was designed to detect a difference of at least 0.8 g (or 8%) in daily salt intake between 2015 and 2010, with 350 volunteers. Based on the response rates in 2010 (16% for the GDPS and 62% for the DCS) and power calculations, an estimation was made on the total number individuals needed to invite. For the random sample from the GDPS, depending on the age category, we expected a response rate between 5% and 20%. And for the random sample from the DCS, we expected a response rate between 30% and 65%.

In 2015, a total number of 2041 individuals were invited to participate (*n* = 1700 aged 19–49 years randomly drawn from the municipal register of Doetinchem, the general Doetinchem population sample (GDPS) and *n* = 341 aged 50–70 years from the Doetinchem cohort study (DCS)). The positive response rate (meaning those willing to participate) was 13% among individuals from the GDPS (*n* = 226) and 52% among DCS participants (*n* = 176). All 402 individuals were invited for one of the instruction meetings during the 3 weeks fieldwork in November 2015. Of those, 333 participants took part in a meeting and started 24-h collection. In total, 328 participants completed the study and handed in their 24-h urine jars at the study center. Upon collection of the jars, 3 individuals appeared to have misinterpreted the protocol (collected multiple days instead of 24-h) and were subsequently excluded from the study population. Furthermore, another 33 participants were excluded due to missing or over-collection of one urine void (based on recorded time of start and finish and completeness in a diary) and one person was excluded due to chronic kidney disease (self-reported in the questionnaire). Finally, two participants were excluded based on creatinine levels, resulting in a total number of 289 participants in the final study population. With this actual sample size of 289 participants, the difference in daily salt intake must have been around 1 g per day to be detected with 80% power.

The study was conducted according to the guidelines laid down in the Declaration of Helsinki. The study protocol was presented to the Medical Ethics Committee of the University Medical Centre Utrecht (15-447/C), who the decided a formal approval was not needed because of the non-invasive character of the study. Written informed consent was obtained from all participants. Participants received a gift voucher (of 30 €) for completing the study.

#### 2.2.2. 24 hr Urine Collection and Assessment of Use of Discretionary Salt

Single 24 h urine collection took place in November 2015 and was performed using the same protocols and procedures as in 2006 and 2010. Participants were provided detailed written and oral instructions at the Municipal Health Centre in Doetinchem by trained researchers. Participants reported the time of start and finish of urine collection, as well as the completeness of the collection in a diary. In addition, a short questionnaire was administered, amongst others, to assess the use of discretionary salt (yes/no) in the week before the collection.

Urinary sodium and creatinine concentration (mmol/L) was determined in each specimen by indirect potentiometry using the Synchron LX system [28,29]. The concentrations (in mmol/L) were multiplied by the total volume of urine to estimate excretion in mmol/day. Conversion from sodium and creatinine in mmol/day to g/day was made by multiplying with the molar mass (Na = 23 g/mol; creatinine = 113 g/mol).

#### 2.2.3. Statistical Analyses

Intake of sodium was calculated by multiplying the excretion with the factor 100/95, which reflects the estimated proportion of intake that is excreted via urine. Sodium intake was converted to salt intake by multiplication with a factor of 2.54.

Incomplete samples, defined as having creatinine excretion ≤5.0 mmol/day or ≤6.0 mmol/day both together with a urine volume of <1 L, and reported missing or overcollection of more than one urine void, were excluded from the analyses (*n* = 38), as well as one subject with chronic kidney disease.

Differences in salt intake between 2006, 2010 and 2015 were assessed by linear regression (using PROC MIXED in SAS 9.3^®^, SAS Institute Inc, Cary, NC, USA, ), correcting for age, education and day of the week.

## 3. Results

### 3.1. Selecting Major Processed Foods Contributing to Salt Intake

**Figure 2:**
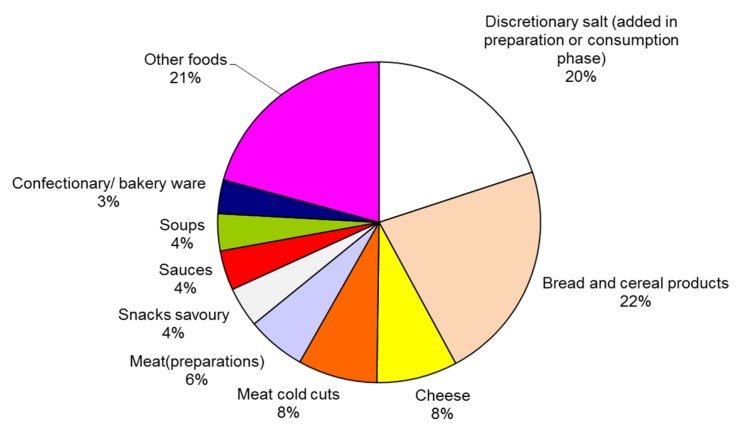
Food groups contributing to salt intake (including discretionary salt) in the Netherlands, food groups contributing more than 3% are included in this study.

Food groups that contributed at least 3% to salt intake and that can be reformulated were included: bread and cereal products, cheese, meat cold cuts and meat (preparations), savoury snacks, sauces, soups, and confectionary and bakery wares (see Figure 2). Together the selected food groups contributed 59% to daily salt intake.

### 3.2. Salt Content of Selected Processed Foods

Salt content of the selected food groups are shown in Table 1 comparing the new 2016 data to the reference in 2011. Table 2 shows the foods with maximum salt levels as set under the agreement of food product improvement and the percentages of compliant foods. Table 3 shows the salt content of foods with maximum salt levels.

The average salt content of “bread” in 2016 was 19% lower than in 2011 (*P* < 0.05; Table 1). From 2011 to 2013, the salt content of “bread” already declined, but since 2013, no further reduction was observed. The salt content of other types of “bread” or “bread replacements” or “breakfast cereal” in 2016 was not significantly different from 2011. The new data for “bread replacements” or “breakfast cereal” however were judged as not representative for major brands.

“Cheese (semi hard and hard-Gouda type)” and “cheese, melted and spreadable” contained less salt in 2016 compared to 2011 (respectively −9%, −13%). However, the differences were not statistically significant. In “meat cold cuts”, in the main underlying subgroups and in “meat preparations” (such as bratwurst, roulades, burger and roast) salt contents in 2016 were not statistically different from 2011. In some selected “meat cold cuts” with set maximum salt levels, lower salt contents were observed. However, none of the changes were statistically significant. In “bacon” and other “meat cold cuts single prepared” (such as ham), the average salt content was respectively 12% and 6% lower in 2016 compared to contents in 2011 (see in Table 3). For “meat products composed prepared” (such as luncheon meat) the salt content was 5% lower, for “filet American” 21% lower. On the other hand for the other “Other cold meats composed raw smoked/dried”, it was 5% higher compared to contents in 2011. Overall, 90% of “meat cold cuts” complied with agreed maximum salt levels (see in Table 2). Within the “savoury snacks” food group, salt contents did not change except for the subgroup “cut potato crisps”. The salt content in this subgroup was 26% lower than in 2011 (*p* < 0.05).

In two of the five subgroups within the food category “sauces”, salt contents were significantly lower. The salt content of “meal sauces on tomatoes/vegetables base” was 15% lower and “meal sauces with a binder” was 19% lower compared to contents in 2011. Although, the data for the latter were judged as not representative for major brands. For “pasta sauces”, “ketchup” and “curry ketchup” maximum salt levels were formulated to be achieved in 2016. “Pasta sauces” contained 15% less salt and “ketchup” 41% less salt compared to 2011 (both *p* < 0.05). For another type of ketchup “curry ketchup”, there was no change in the salt content, although 93% of this type of ketchup complies with current targets (Table 2). The salt content of the “soups sold as liquid” was statistically significantly lower (by 12%) in the newly submitted data compared to 2011. There was no statistically significant change observed in the salt content of “soups instant” (composition as prepared). For the entire group of “soups” (sold as liquid and instant), the salt content was 9% lower compared to 2011 (*p* < 0.05).

“Processed vegetable and legumes” contributed less than 3% to the average daily salt intake. The sector agreed on salt reduction targets. In the subgroup “green peas, carrots, peas/beans and carrots” the salt content was 25% lower than in 2011. This difference was statistically significant. In the group “butter beans, haricots, mushrooms“the salt content was 37% lower than in 2011, but this is not statistically significant. For the processed legumes the average salt content was significantly lower (42%) compared to 2011.

### 3.3. Estimated Daily Salt Intake from 24 h Urinary Salt Excretion

**Figure 3:**
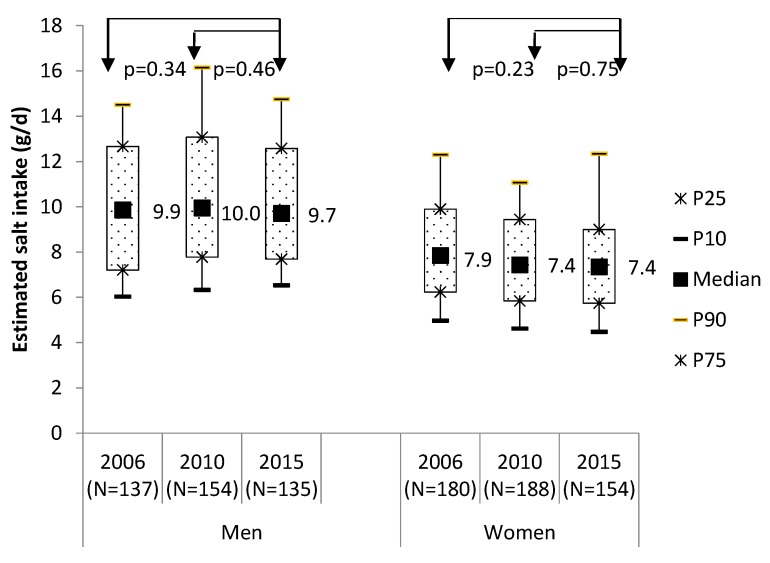
Salt intake (g per day) estimated from 24 hr urine excretion in 2006, 2010 and 2015, correcting for age, education and day of the week. The proportion of participants reporting the use of discretionary salt was lower in 2010 as compared to 2006 (81% in 2010 versus 88% in 2006; *p* = 0.009). In 2015, the use of discretionary salt (83%) was not statistically significant different compared to 2010 (*p* = 0.46).

The 24 h urinary salt excretion was similar in 2006, 2010 and 2015. In men, median estimated salt intake was 9.7 g/day in 2015. This was not different from 2006 (9.9 g/day; *p* = 0.34) and 2010 (10.0 g/day in 2010; *p* = 0.46). In women, median salt intake was 7.4 g/day in 2015. Compared to 2006 (7.9 g/day; *p* = 0.23) and 2010 (7.4 g/day; *p* = 0.75), see Figure 3.

## 4. Discussion

This study showed that the salt content of certain foods in the Netherlands, such as bread, certain sauces, soups, potato crisps, and processed legumes and vegetables, has been reduced over the period 2011–2016. Significant salt reductions found ranged from −12% (some soups), to −19% (bread), and −42% (for processed legumes) in 2016 compared to 2011. In other food groups, such as meat cold cuts and cheese, the salt contents were not significantly different.

Salt reduction targets for foods can be voluntary or mandatory. In the Netherlands, mandatory maximum levels are set for bread (requested by the sector itself) and voluntary for other food categories without formal sanctioning. In some food categories, such as for processed vegetables/legumes and for ketchups, considerable salt reduction was achieved (for example −42% for processed legumes and −41% for ketchup) with the voluntary commitments. In other food categories, the voluntary set salt maximum levels showed limited ambitions and/or did not lead to significantly reduced salt contents. For example, in some of the meat cold cuts subcategories the observed changes were small and not statistically significant.

Globally, bread is the most targeted food for salt reduction, followed by foods such as other bakery products, processed meats, sauces and convenience meals [30]. The majority (81%) of national salt reduction strategies, like in the Netherlands, include industry engagement to reduce the salt content of foods [30]. To date there is little scientific knowledge concerning the factors that influence the effectiveness of public-private agreements to reach a certain societal goal. Bryden et al. [31] evaluated voluntary agreements between business and government trying to identify factors of success. They concluded that, if properly implemented and monitored, voluntary agreements can be effective and business can help to achieve public policy aims. Proper implementation includes realistic, but stretching, targets for businesses to achieve real changes [31]. In addition, substantial disincentives for non-participation and costly sanctions for non-compliance seem to improve the effectiveness of agreements [31]. Further research should evaluate whether these advices also apply to the field food product improvement.

The ultimate goal of lowering salt contents of foods is to reduce salt intake at the population level, and in this way improve health. To reach current salt intake reduction targets via food product improvement alone, all major salt contributing foods contributing need to be lowered by 30−40% [12]. Although this is an ambitious target, it might be possible for food categories such as bread, meat cold cuts, cheese, and soup as is shown via salt-reduced foods as used in experimental settings [32,33,34]. In these experiments, the participants consumed and liked the salt reduced foods similarly to the regular salted foods. However, in isolation, reformulation is unlikely to provide a complete solution to the challenge of improving eating patterns and nutrient provision, although it is a contributor [35]. More likely to be effective is a combination of strategies of food reformulation with changed food choice behaviour (e.g., more vegetables and less meat/cheese, choosing meat cold cuts with low salt contents, reducing discretionary salt) and/or reduction of portion sizes.

Athough we observed salt reduction in some food categories, median salt intake between 2006 and 2015 did not differ using 24 hr urinary analysis. Only a limited number of countries observed significant decreased salt intakes at the population level [11]. The significant decrease per day ranged from −1.15 (95% confidence interval (CI) −1.69 to −0.61) G/day in Finland [36], −0.9 G/day in the United Kingdom (UK) [37] to −0.35 (95% CI −0.52 to −0.18) G/day in Ireland [11]. The countries with successful salt reductions at the population level started at relatively high levels—11.8 g/day [36] in Finland and 9.5 g per day in UK [37], compared to 8.7 G/day in 2006 in our study population. In line with our own results, several countries, for example Austria and Switzerland, did not show a statistically significant change in salt intake (G/day) from pre- to post-intervention [11].

Recent reviews assessed the effectiveness of different types of population-level interventions implemented by governments for dietary salt reduction [11,30]. Especially, a multi-component approach is needed to reduce daily salt intakes, including more than one intervention activity [11]. In addition, incorporation of initiatives of a structural nature is needed (such as food product reformulation or the availability of low salt foods via public procurement). Seventy-five countries now have a national salt reduction strategy [30,38]. The majority of the strategies are to a certain extent multicomponent, as they include industry engagement to reformulate products (*n* = 61), establishment of salt content targets for foods (39), consumer education (71), front-of-pack labelling schemes (31), taxation on high-salt foods (3), and interventions in public institutions (54) [30]. The countries observing a statistically significant salt reduction had a multicomponent approach. In the UK, many activities were undertaken to lower salt intake from 2003 onwards. Efforts included public information/education campaigns, on-package nutrition information, restrictions on marketing to children, food procurement policy in specific settings, and food product reformulation [11,39]. Finland introduced mandatory warning labels on high salt foods in addition to food reformulation activities [40,41]. Furthermore, a long breath is needed to achieve salt reduction. In the UK it took more than 10 years, and in Finland 20 years, to achieve small steps in salt reduction in the population. In the Netherlands, the approach focusses on food reformulation activities and is ongoing. Additional product category agreements on salt reduction are planned for 2017, for example for meat products and breakfast cereals. The effect on daily salt intake is also dependent on food consumption patterns. Recent small changes in food consumption patterns in the Netherlands may either be in line with (e.g., lower meat consumption), or cancelling (e.g., more sauces consumption) the impact of recent modest reductions in the salt content of processed food [42].

### Limitations and Strengths

The strength of our study is that we made a structured, comprehensive comparison of salt contents of foods, including the major contributors to daily intake. For many foods, data of independent chemical analyses were provided by the Dutch Food Safety Authority. The analytical data were complemented with nutritional label information. A representativeness check of our data with respect to the presence of major (private and supermarket) brands was carried out. Salt content stated on food labels may be calculated from food composition tables and differs from contents derived from chemical analyses. In most food categories, salt contents given on labels are higher than contents based on chemical analyses [26]. With increasing use of label information based on calculations from food composition tables, reduced salt content may be missed, because of circular reasoning. It therefore remains important that (a sample of) data are checked regularly by independent chemical analyses. To effectively monitor changes in food composition, researchers should ideally have access to food composition data of all individual foods sold in retail for multiple years. Examples of such a monitoring system exist from the USA [43,44] and France [45]. Food groups would a priori be comparable over subsequent years, as all foods (adjusted for marked share) are represented, and external validity is most likely higher than in the current study. Data quality needs to be taken into account, as indicated above. The use of generic food composition data (NEVO 2011) as a reference poses some issues. It is intrinsically difficult to compare generic foods with individual foods, as they are by definition different. The generic foods used as the reference were aggregated from food composition data of individual foods obtained over a period of several years, thus with less variation in salt contents than an analyses based on individual foods.

For the salt intake based on 24 hr urine, power calculations showed that this study had sufficient power to detect a reduction of around 1 g in daily salt intake in the total population between 2010 and 2015, given the sample size in 2015 of 289 subjects. To comply with the recommendations of 6 g a day 30–40% salt reduction is needed in major salt contributing foods. In order to detect smaller reductions (of 4–5%) in the mean population intake of salt, which were more likely given the current achievements, would have needed a larger sampling size. Thus, the fact that no changes in population intake were found despite salt reduction in some foods is not in contradiction with each other. Participants for the 24 hr urine study were sampled from a single town in the Netherlands and the participation rate was low; this limits the generalizability of the results from our study to the general population. Compared to the general Dutch population, participants were more likely to be non-smokers and were higher educated. However, as the study characteristics of the study populations over the three years were highly comparable, this study design may demonstrate a trend in salt intake.

## 5. Conclusions

In the Netherlands, the salt content of bread, certain sauces, soups, potato crisps, and processed legumes and vegetables has been reduced over the period 2011–2016. However, median salt intake in 2006 and 2015 using 24 hr urinary analysis remains well above the recommended intake of 6 g.

## Figures and Tables

**Table 1:** Salt contents of foods (g/100 g) in 2016 compared to 2011, main food categories.

		Reference 2011-Salt Content				New Data 2016-Salt Content			Difference	
Food Group		*N*	Uval	AVG	SD	*n*	AVG	SD	(%)	
**Bread and cereal products**										
	Bread ◊	25	82	1.29	0.24	194	1.04	0.13	−9%	¥
	Bread luxury natural and sweet ^b^	11	37	0.97	0.17	41	0.90	0.17	−7%	
	Bread replacements ^b^	21	2	1.23	0.59	61	1.23	0.60	0%	
	Breakfast cereal ^b^	24	2	0.60	0.54	76	0.51	0.48	−15%	
**Cheese**										
	Cheese, semi hard and hard ◊	18	26	2.04	0.41	175	1.87	0.40	−9%	
	Cheese, melted and spreadable ^b^	12	2	1.52	0.52	73	1.33	0.49	−13%	
**Meat cold cuts**										
	Single prepared ◊	13	8	2.54	0.58	153	2.36	0.55	−7%	
	Composed prepared ◊	23	7	2.20	0.25	250	2.10	0.33	−5%	
	Single raw smoked/dried ^b^	5	9	3.92	0.87	44	3.96	1.36	1%	
	Composed raw smoked/dried ◊	9	7	3.15	0.58	105	3.04	0.90	−4%	
**Meat(preparations)**										
	Meat preparations unprepared	14	10	1.56	0.62	111	1.83	0.56	18%	
**Snacks savoury**										
	Ragout snack (“kroket” type)	1	6	1.40		53	1.32	0.34	−6%	
	Snacks savoury with meat	2	5	1.78	0.17	28	1.88	0.35	5%	
	Cut potato crisps	8	7	1.75	0.49	37	1.29	0.28	−26%	¥
	Pelleted crisps	9	5	2.18	0.85	91	2.06	0.79	−6%	
	Salted biscuits	6	6	2.15	1.20	31	2.22	0.58	3%	
**Sauces**										
	Tomato/vegetable meal sauces *^,^◊	38	7	1.13	0.27	87	0.96	0.41	−15%	¥
	Tomato/vegetable based cold sauces ◊	8	3	2.19	0.60	115	1.94	0.84	−11%	
	Emulsion based sauces	15	4	1.53	0.37	121	1.40	0.56	−8%	
	Sauces, peanut *	10	4	1.70	0.35	16	1.50	0.50	−12%	
	Meal sauces with a binder *^,b^	40	10	1.35	0.48	36	1.09	0.30	−19%	¥
**Soups ◊**										
	Soups sold as liquid *	48	7	0.89	0.23	109	0.78	0.12	−12%	¥
	Soups instant prepared *	28	7	0.90	0.24	52	0.87	0.17	−3%	
**Confectionary and bakery ware**										
	Cakes ^b^	4	4	0.79	0.44	51	0.75	0.35	−4%	
	Biscuits ^b^	28	3	0.57	0.28	24	0.77	0.18	36%	¥
	Shortbreads	7	11	0.80	0.28	68	0.60	0.29	−25%	
	Pies and pastries (sweet) ^b^	9	8	0.44	0.17	70	0.35	0.21	−20%	

*N* = number of generic foods codes, UVal = average number of underlying values per generic food. *n* = number of new data points; ¥ Statistically significant difference (*p* < 0.05) between 2016 and 2011; * Food composition data are supplemented with Innova data on food composition; ^a^: Information of less than 50% of supermarket brands is available in the new data; ^b^: Information of less than 50% of other major brands (excluding supermarket brands) is available in the new data; ◊ Food with salt reduction targets (see Table 2).

**Table 2:** Foods with maximum salt levels agreed on via Agreement on Improvement of Product Composition.

Food Group		Food with Maximum Salt Targets	Maximum	Start and End Date	<Max Salt Level
			g/100 g		% of Foods
**Bread and cereal products**				2010–01/01/2013	
	Bread	White, brown, wholemeal, multigrain bread; both large and small and baguette	1, 8% ^‡^		n.a.
**Cheese**				2010–/12/2015	
	Cheese	Gouda cheese 48+	−10% ^∏^		
**Meat cold cuts**				06/2013–06/2015	90%
	Single prepared	Bacon, grilled	2.80		
		Others	2.54		
	Composed prepared		2.36		
	Composed raw smoked/dried	Filet American	2.25		
		Others	3.20		
**Sauces**				01/01/2015–30/06/2016	
	Tomato/vegetable meal sauces	Sauce for pasta	1.30		96%
	Tomato/vegetable based cold sauces	Ketchup	2.49		87%
		Curry ketchup	2.06		93%
**Soups**				01/01/2015–30/06/2016	
	Soups sold as liquid and instant prepared	Soups	0.89		79%
**Processed vegetables and legumes**				2011–2013	n.a.
	Processed vegetables	Peas and/or carrots, bean	0.38		
		Butter beans, haricots, mushrooms	0.46		
	Processed legumes	Legumes	0.51		

^‡^ The maximum 1.8% salt (based on dry matter), with an average bread moisture content of 64% which is around 1.15 g salt per 100 g bread; ^∏^ The target for cheese is 10% average reduced salt content, no maximum defined; n.a. not available; and percentage of foods below maximum in 2016 (adapted from reference [27]).

**Table 3:** Salt contents of foods with salt reduction targets (g/100 g) in 2016 compared to 2011.

Food Group	Food with Maximum Salt Targets	Reference 2011-Salt Content				New Data 2016-Salt			Difference	
		*N*	Uval	AVG	SD	*n*	AVG	SD	(%)	
**Bread and cereal products**										
Bread	White, brown, wholemeal, multigrain bread; both large and small and baguette	19	99	1.27	0.27	161	1.02	0.11	−21%	¥
**Cheese**										
Cheese	Gouda cheese 48+	7	58	2.09	0.35	80	1.89	0.35	−11%	
**Meats cold cuts**										
Single prepared	Bacon, grilled	2	3	2.76	1.41	26	2.46	0.83	−12%	
	Others	11	9	2.45	0.42	127	2.34	0.47	−6%	
Composed prepared		23	5	2.17	0.25	250	2.10	0.33	−5%	
Composed raw smoked/dried	Filet American	1	13	2.26	0.00	25	1.82	0.32	−21%	
	Others	8	7	3.21	0.51	80	3.42	0.64	5%	
**Sauces**										
Tomato/vegetable meal sauces	Sauce for pasta	38	7	1.12	0.26	87	0.96	0.41	−15%	¥
Tomato/vegetable based cold sauces	Ketchup	2	5	2.63	0.53	18 ^a^	1.58	0.36	−41%	¥
	Curry ketchup	1	2	1.69	0.00	19	1.69	0.41	−1%	
**Soups**										
Soups sold as liquid and instant prepared		76		0.89	0.23	204	0.82	0.17	−9%	¥
**Processed vegetables and legumes**										
Processed vegetables	Peas and/or carrots, bean	4		0.47	0.12	44	0.35	0.10	−25%	¥
	Butter beans, haricots, mushrooms	2		0.64	0.02	17	0.41	0.16	−37%	
Processed legumes	Legumes	2	1	0.88	0.32	21	0.52	0.13	−42%	¥

*N* = number of generic foods codes, UVal = average number of underlying values per generic food. *n* = number of new data points; ¥ Statistically significant difference (*p* < 0.05) between 2016 and 2011; ^a^: Exclusion of one ketchup because salt content was lower than 0.05 g/100 g.

## References

[1] Global Burden of Disease, Risk Factors Collaborators (2015). Global, regional, and national comparative risk assessment of 79 behavioural, environmental and occupational, and metabolic risks or clusters of risks in 188 countries, 1990–2013: A systematic analysis for the Global Burden of Disease Study 2013. Lancet.

[2] World Health Organization (2013). Global action plan for the prevention and control of noncommunicable diseases 2013–2020.

[3] Kromhout D., Spaaij C.J., de Goede J., Weggemans R.M. (2016). The 2015 Dutch food-based dietary guidelines. Eur. J. Clin. Nutr..

[4] Hendriksen M.A., van Raaij J.M., Geleijnse J.M., Wilson-van den Hooven C., Ocke M.C., van der A D.L. (2014). Monitoring salt and iodine intakes in Dutch adults between 2006 and 2010 using 24 h urinary sodium and iodine excretions. Public Health Nutr..

[5] Van Rossum C.T.M., Fransen H.P., Verkaik-Kloosterman J., Buurma-Rethans E.J.M., Ocke M.C. (2011). Dutch National Food Consumption Survey 2007–2010. Diet of Children and Adults Aged 7 to 69 Years. RIVM Rapport 350050006.

[6] Micha R., Khatibzadeh S., Shi P., Fahimi S., Lim S., Andrews K.G., Engell R.E., Powles J., Ezzati M., Mozaffarian D. (2014). Global, regional, and national consumption levels of dietary fats and oils in 1990 and 2010: A systematic analysis including 266 country-specific nutrition surveys. BMJ.

[7] Powles J., Fahimi S., Micha R., Khatibzadeh S., Shi P., Ezzati M., Engell R.E., Lim S.S., Danaei G., Mozaffarian D. (2013). Global, regional and national sodium intakes in 1990 and 2010: A systematic analysis of 24 h urinary sodium excretion and dietary surveys worldwide. BMJ Open.

[8] Temme E.H.M., Westenbrink S., Toxopeus I.B., Hendriksen M.A.H., Werkman A.M., Klostermann V.L.C. (2013). Natrium en Verzadigd Vet in Beeld [Sodium and Saturated Fat Content of Foods]. RIVM Briefrapport 350022002.

[9] Auestad N., Hurley J.S., Fulgoni V.L., Schweitzer C.M. (2015). Contribution of Food Groups to Energy and Nutrient Intakes in Five Developed Countries. Nutrients.

[10] World Health Organisation (2013). Global Action Plan for the Prevention and Control of Noncommunicable Diseases 2013–2020.

[11] McLaren L., Sumar N., Barberio A.M., Trieu K., Lorenzetti D.L., Tarasuk V., Webster J., Campbell N.R. (2016). Population-level interventions in government jurisdictions for dietary sodium reduction. Cochrane Database Syst. Rev..

[12] Hendriksen M.A., Verkaik-Kloosterman J., Noort M.W., van Raaij J.M. (2015). Nutritional impact of sodium reduction strategies on sodium intake from processed foods. Eur. J. Clin. Nutr..

[13] McLaren L., McIntyre L., Kirkpatrick S. (2010). Rose’s population strategy of prevention need not increase social inequalities in health. Int. J. Epidemiol..

[14] Actieplan Zout in Levensmiddelen (2008). Action Plan. Salt in Foods.

[15] Besluit van 15 november 2012, houdende wijziging van het Warenwetbesluit Meel en brood inzake het maximale zoutgehalte van brood. Staatsblad van het Koninkrijk der Nederlanden, nr 598, Den Haag, 2012. Commodities Act with Regulation on the Maximum Level of Salt in Bread. https://zoek.officielebekendmakingen.nl/stb-2012-598.html.

[16] Vijfde Landelijke Steekproef Zoutgehalte in Brood, maart–mei 2013 (Fifth annual sampling of salt content of bread, March-May 2013).

[17] Zesde Landelijke Steekproef Zoutgehalte in Brood, februari–april 2015 (Sixth annual sampling of salt content of bread, February-April 2015).

[18] (2014). Akkoord Verbetering Productsamenstelling Zout, Verzadigd Vet, Suiker (Calorieën) (National agreement o Improve Product Composition: Salt, Saturated Fat, Sugar (Calories)). http://www.akkoordverbeteringproductsamenstelling.nl/en.

[19] Temme E.H.M., Milder I.E.J., Westenbrink S., Toxopeus I.B., Van den Bogaard C.H.M., Van Raaij J.M.A. (2015). Monitoring Productsamenstelling Voor Zout, Verzadigd Vet en Suiker. RIVM Herformuleringsmonitor 2014. RIVM Briefrapport 2015–0034 (Monitoring Product Composition for Salt, Saturated Fatty Acid and Sugar. RIVM Reformulation Monitor 2014, RIVM Letter Report 2015–0034).

[20] (2011). NEVO-online (Nederlands Voedingsstoffenbestand), NEVO-online versie 2011. (Dutch Food Composition Database NEVO-online version 2011).

[21] Website Akkoord Verbetering Productsamenstelling (Website National Agreement to Improve Product Composition). http://www.akkoordverbeteringproductsamenstelling.nl.

[22] Voedingscentrum (2011). Richtlijnen Voedselkeuze.

[23] Innova Innova’s Food & Beverage Database. http://www.innovadatabase.com/home/index.rails.

[24] Voedingscentrum Levensmiddelendatabank (LEDA) (Food Database LEDA) http://www.voedingscentrum.nl/professionals/productaanbod-en-levensmiddelendatabank.aspx.

[25] (2016). Monitoring Van Het Gehalte Aan Keukenzout in Diverse Levensmiddelen 2015 (Monitoring the Content of Salt in Several Foods 2015).

[26] NVWA (2017). Monitoring Van Het Keukenzoutgehalte in Diverse Levensmiddelen 2016 (Monitoring the Content of Salt in Several Foods 2016).

[27] (2017). Monitoring Van Het Keukenzout- en Verzadigd Vetgehalte in Levensmiddelen Waarvoor Afspraken Zijn Gemaakt in Het Akkoord Verbetering Productsamenstelling 2016 (Monitoring the Content of Salt and Saturated Fatty Acids in Several Foods with Reduction Targets 2016).

[28] Chemistry Information Sheet CREm Creatinine. Beckman Coulter Synchron LX System(s). www.beckmancoulter.com.

[29] Chemistry Information Sheet NA Sodium. Beckman Coulter Synchron LX System(s). www.beckmancoulter.com.

[30] Trieu K., Neal B., Hawkes C., Dunford E., Campbell N., Rodriguez-Fernandez R., Legetic B., McLaren L., Barberio A., Webster J. (2015). Salt Reduction Initiatives around the World—A Systematic Review of Progress towards the Global Target. PLoS ONE.

[31] Bryden A., Petticrew M., Mays N., Eastmure E., Knai C. (2013). Voluntary agreements between government and business—A scoping review of the literature with specific reference to the Public Health Responsibility Deal. Health Policy.

[32] Willems A.A., van Hout D.H., Zijlstra N., Zandstra E.H. (2014). Effects of salt labelling and repeated in-home consumption on long-term liking of reduced-salt soups. Public Health Nutr..

[33] Bolhuis D.P., Temme E.H., Koeman F.T., Noort M.W., Kremer S., Janssen A.M. (2011). A salt reduction of 50% in bread does not decrease bread consumption or increase sodium intake by the choice of sandwich fillings. J. Nutr..

[34] Janssen A.M., Kremer S., van Stipriaan W.L., Noort M.W., de Vries J.H., Temme E.H. (2015). Reduced-sodium lunches are well-accepted by uninformed consumers over a 3-week period and result in decreased daily dietary sodium intakes: A randomized controlled trial. J. Acad. Nutr. Diet..

[35] Buttriss J.L. (2013). Food reformulation: The challenges to the food industry. Proc. Nutr. Soc..

[36] Laatikainen T., Pietinen P., Valsta L., Sundvall J., Reinivuo H., Tuomilehto J. (2006). Sodium in the Finnish diet: 20-Year trends in urinary sodium excretion among the adult population. Eur. J. Clin. Nutr..

[37] Shankar B., Brambila-Macias J., Traill B., Mazzocchi M., Capacci S. (2013). An evaluation of the UK Food Standards Agency’s salt campaign. Health Econ..

[38] Trieu K., McLean R., Johnson C., Santos J.A., Raj T.S., Campbell N.R.C., Webster J. (2016). The Science of Salt: A Regularly Updated Systematic Review of the Implementation of Salt Reduction Interventions (November 2015 to February 2016). J. Clin. Hypertens..

[39] He F.J., Brinsden H.C., MacGregor G.A. (2014). Salt reduction in the United Kingdom: A successful experiment in public health. J. Hum. Hypertens..

[40] Pietinen P., Valsta L.M., Hirvonen T., Sinkko H. (2008). Labelling the salt content in foods: A useful tool in reducing sodium intake in Finland. Public Health Nutr..

[41] WHO (2013). Mapping Salt Reduction Initiatives in the WHO European Region.

[42] Van Rossum C., Buurma-Rethans E., Vennemann F., Beukers M., Brants H., de Boer E., Ocké M. (2016). The Diet of the Dutch : Results of the First Two Years of the Dutch National Food Consumption Survey 2012–2016.

[43] Gillespie C., Maalouf J., Yuan K., Cogswell M.E., Gunn J.P., Levings J., Moshfegh A., Ahuja J.K., Merritt R. (2015). Sodium content in major brands of US packaged foods, 2009. Am. J. Clin. Nutr..

[44] Poti J.M., Dunford E.K., Popkin B.M. (2017). Sodium reduction in US households’ packaged food and beverage purchases, 2000 to 2014. JAMA Intern. Med..

[45] Menard C., Dumas C., Goglia R., Spiteri M., Gillot N., Combris P., Ireland J., Soler L.G., Volatier J.L. (2011). OQALI: A French database on processed foods. J. Food Compos. Anal..

